# Effects of 1-*N*-Naphthylphthalamic Acid on Root and Leaf Development of *Muscari armeniacum* and the Related Metabolic and Physiological Features

**DOI:** 10.3390/ijms26136431

**Published:** 2025-07-03

**Authors:** Agnieszka Marasek-Ciołakowska, Aleksandra Machlańska, Wiesław Wiczkowski, Dorota Szawara-Nowak, Lesław B. Lahuta, Justyna Góraj-Koniarska, Kensuke Miyamoto, Junichi Ueda, Marian Saniewski, Marcin Horbowicz

**Affiliations:** 1 The National Institute of Horticultural Research, Konstytucji 3 Maja 1/3, 96-100 Skierniewice, Poland; agnieszka.marasek@inhort.pl (A.M.-C.); aleksandra.machlanska@inhort.pl (A.M.); justyna.goraj@inhort.pl (J.G.-K.); marian.saniewski@inhort.pl (M.S.); 2 Institute of Animal Reproduction and Food Research of the Polish Academy of Sciences, Biotransformation and Bioavailability of Phytochemicals Team, Trylińskiego 18, 10-683 Olsztyn, Poland; w.wiczkowski@pan.olsztyn.pl (W.W.); d.szawara-nowak@pan.olsztyn.pl (D.S.-N.); 3 Department of Plant Physiology, Genetics and Biotechnology, University of Warmia and Mazury, Oczapowskiego 1A, 10-719 Olsztyn, Poland; lahuta@uwm.edu.pl; 4 Faculty of Liberal Arts, Sciences and Global Education, Osaka Metropolitan University, 1-1 Gakuen-cho, Naka-ku, Sakai 599-8531, Japan; k.miyamoto@omu.ac.jp; 5 Department of Biological Science, Graduate School of Science, Osaka Prefecture University, 1-1 Gakuen-cho, Naka-ku, Sakai 599-8531, Japan; w21913n@omu.ac.jp

**Keywords:** *Muscari armeniacum*, bulbs, 1-*N*-naphthylphthalamic acid, root swelling, gravitropic response, organic acids, carbohydrates, phenolic compounds

## Abstract

The effects of 1-*N*-naphthylphthalamic acid (NPA) applied as an aqueous solution on uncooled grape hyacinth (*Muscari armeniacum*) bulbs were investigated, focusing on histological measurements and the determination of various metabolites in developing roots. *M. armeniacum* bulbs were kept for a defined number of days in distilled water (control) or aqueous NPA solutions, and then 2 cm sections of root tips were taken for histological measurements. Longitudinal and cross sections were taken in these root pieces, followed by measurements of their basic parts and microscopic images. Determinations of polar compounds by GC/MS and phenolic metabolites by HPLC/MS/MS were carried out in freeze-dried root samples. NPA inhibited the growth of the roots and caused swelling of their elongation parts, as well as changes in the dimensions of other parts of the roots and disruption of the gravitropic direction of their growth. However, NPA did not affect leaf growth and the amino acid, organic acid, and major carbohydrate content in the roots, but increased the level of unknown saccharides, probably oligofructans. The decrease in the contents of many phenolic compounds observed in our study under the influence of NPA may indicate that this could be one of the symptoms/causes of root growth disorders. In turn, the reduction in polyphenol levels may have been related to an increase in the number and length of root hairs.

## 1. Introduction

Grape hyacinth (*Muscari armeniacum* Leichtl. ex Baker) is one of the important ornamental plants grown worldwide [1,2]. The cooling stage of *M. armeniacum* bulbs is essential for the growth of its inflorescence and flowering, but does not affect leaf growth [3]. Leaf and inflorescence stem growth in *M. armeniacum* is controlled by a set of plant growth regulators. The application of gibberellic acid (GA), benzyladenine (BA), and their mixture to *M. armeniacum* bulbs did not affect leaf growth [3]. In contrast, abscisic acid (ABA) inhibited leaf growth. However, neither GA, BA, nor ABA interrupts inflorescence stem dormancy in uncooled *M. armeniacum* bulbs. In addition, indolyl-3-acetic acid (IAA) applied at the site of the removed inflorescence bud induced inflorescence stem growth [3].

The herbicidal action of 1-*N*-naphthylphthalamic acid (NPA) was discovered in the 1940s, and NPA was soon used as an herbicide called Naptalam [4]. Naptalam is a selective pre-emergence pesticide that controls many broadleaf weeds and some grasses [5]. It has been sold under the tradenames Alanap-L and Rescue in the United States, as Alanap-3 in Canada, and as Alanap-R in Australia, and used in the cultivation of soybeans, peanuts, cucumbers, melons, maize, cranberries, and ornamentals [5,6,7,8].

NPA was also shown to be an inhibitor of polar auxin transport in plant tissues due to its binding to PIN auxin transporters [9,10,11,12]. Results of our previous study showed that the application of NPA to the stem of *Solidago canadensis* caused abnormal radial growth of the stem, changes in its histological structure, as well as leading to the accumulation of IAA and disturbing its metabolism [13]. A similar phenomenon of NPA-induced stem swelling and accumulation of IAA occurred in *Bryophyllum calycinum* [14]. Additionally, NPA applied to tomato seedlings inhibited root growth and stimulated hypocotyl elongation, and the epidermal cells were more elongated and narrower than in control seedlings [15].

Auxin is transported by the vascular system from the shoot apex to the root apex and is redistributed to cortical and epidermal tissues. It is then transported to the basal regions of the root, where it regulates cell division and elongation, as well as root hair formation [16]. A non-uniform distribution of auxin in the plant is a direct cause of gravity-related growth disorders [17]. Several other studies have also shown the inhibition of polar auxin transport and rooting in plants after NPA application [18,19,20,21]. Additionally, Akhami et al. [22] showed that the application of NPA to petunia seedlings inhibited adventitious root formation. As a result, the glucose, fructose, and sucrose contents in the stems were much higher compared to those in untreated seedlings. These authors believe that this was due to the effect of NPA inhibiting root growth. Also, NPA completely inhibited rooting in grape explants [23] and reduced soybean leaf growth as a result of reduced cell division [24].

The application of the NPA to the roots of *Arabidopsis thaliana* blocked the gravity response, root waving, and root elongation [25,26]. Gravity sensing takes place in the root cap cells, where sedimentation of starch-filled amyloplasts triggers a pathway that results in the relocation to the lower half of the cell of PIN proteins that facilitate auxin efflux [27]. This causes auxin accumulation in the lower half of the root and bending of the root tip in the elongation zone [27,28].

Flower bulbs contain reserve carbohydrates such as fructans, starch, and other polysaccharides, as well as monosaccharides and sucrose, which play an important role during germination and initial root and leaf growth [29,30]. Fructans are remobilized during the germination or growth of various plants [31,32,33,34,35]. According to Ranwala and Miller [29], fructans in *M. armeniacum* bulbs accounted for 36.2% of their dry weight, while starch content reached 24.4%. Fructans in bulbs of this species contained glucose and fructose moieties in a ratio of 1 to 26 [31,32]. Further analyses have shown that it belongs to mixed-linkage type fructans, containing both the inulin and the levan type structures. Furthermore, preliminary results showed that, in addition to fructans and starch, *M. armeniacum* bulbs also contained acidic arabinogalactans [33,34,35]. These stored carbohydrates might be remobilized during early growth, so we investigated whether NPA treatment alters carbohydrate utilisation in the roots. Additionally, we investigated the content of other carbohydrates whose changes in content may indicate their involvement in the biosynthesis of other metabolites.

The current study is a continuation of our previous investigations in which NPA was applied to the stems of *Solidago canadensis* and *Bryophyllum calycinum* plants [13,14]. In the present study, we investigated the effects of NPA on the growth of roots and leaves developing from uncooled *M. armeniacum* bulbs, focusing on changes in the histological structures of the roots, as well as the content of polar and phenolic metabolites. To our knowledge, this has not been previously examined in *M. armeniacum* bulbs.

## 2. Results

### 2.1. The Morphology and Histological Measurements of M. Armeniacum Roots

**Figure 1:**
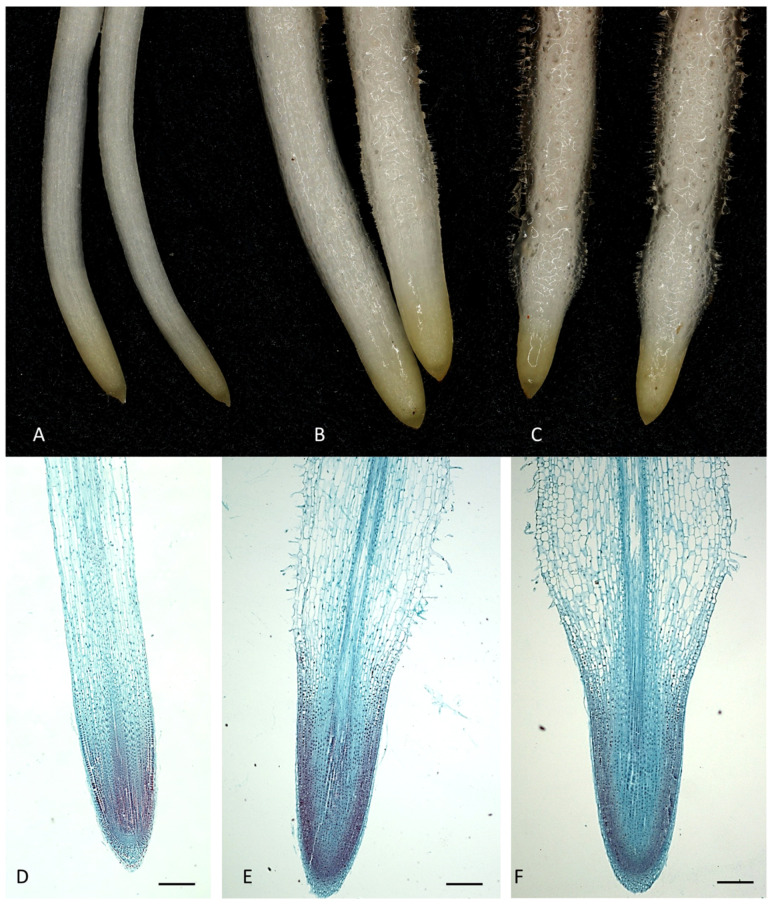
Effect of NPA on root morphology in uncooled *M. armeniacum* bulbs after a 9-day treatment period. (**A**–**C**) Phenotype in stereoscopic microscope; (**A**) roots grown in water (control); (**B**) roots treated with 5 mg/L of NPA; (**C**) roots treated with 10 mg/L of NPA; (**D**–**F**) longitudinal-section of the root in light microscope; (**D**) root grown in water (control); (**E**) root treated with 5 mg/L of NPA; (**F**) root treated with 10 mg/L of NPA. Bars represent 200 µm.

**Figure 2:**
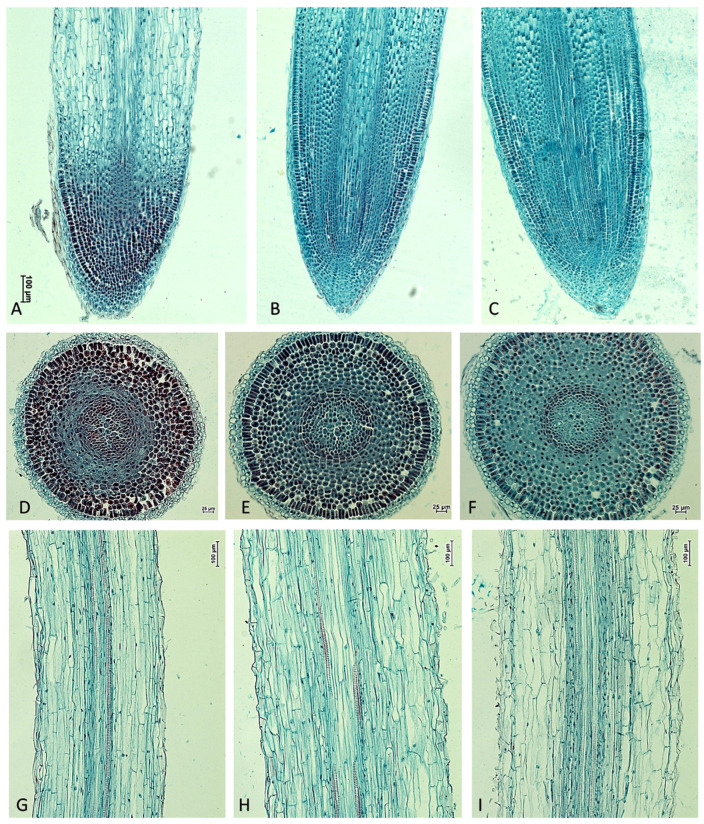
The effect of NPA application on anatomy of roots developed in uncooled *M. armeniacum* bulbs after 9-day treatment period. (**A**–**C**) Longitudinal section; (**D**–**F**) apical meristem cross section; (**G**–**I**) longitudinal section of the root of elongation zone. (**A**,**D**,**G**) Roots developed in water (control). (**B**,**E**,**H**) Roots developed in aqueous solution of NPA at 5 mg/L. (**C**,**F**,**I**) Roots developed in aqueous solution of NPA at 10 mg/L.

**Figure 3:**
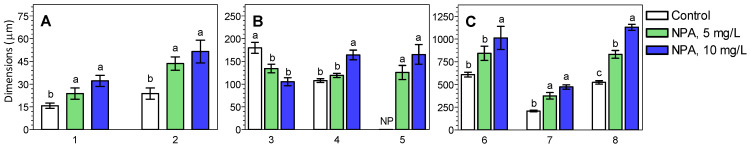
Results of measurements of different parts of the *M. armeniacum* root whose bulbs were kept in water or aqueous NPA solutions. Description of measured part of root: (**A**): 1—Thickness of cortex cell; 2—Thickness of the rhizodermis/epiblema; (**B**): 3—Cap thickness; 4—Width of stele; 5—Length of root hairs; NP means not present; (**C**): 6—Thickness of growth cap; 7—Width of cortex; 8—Root thickness. The results (bars ± SD) of the measurements of the respective root parts marked with the same letter do not differ from each other at a significance level of *p* = 0.05 according to Duncan’s test.

**Figure 4:**
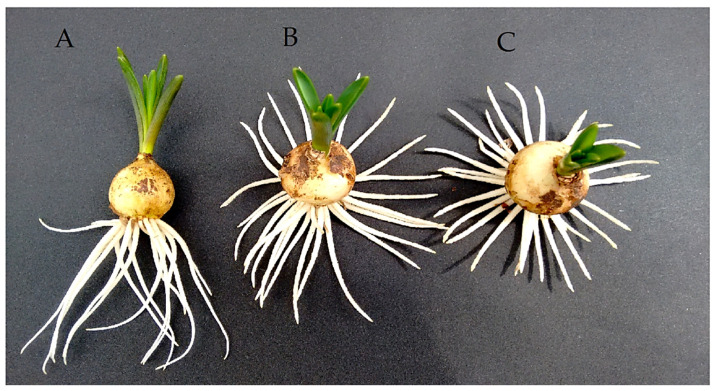
Roots developed from *M. armeniacum* bulbs kept for 17 days in water (**A**) or bulbs kept in an aqueous solution of NPA at 5 mg/L (**B**) or 10 mg/L (**C**).

**Figure 5:**
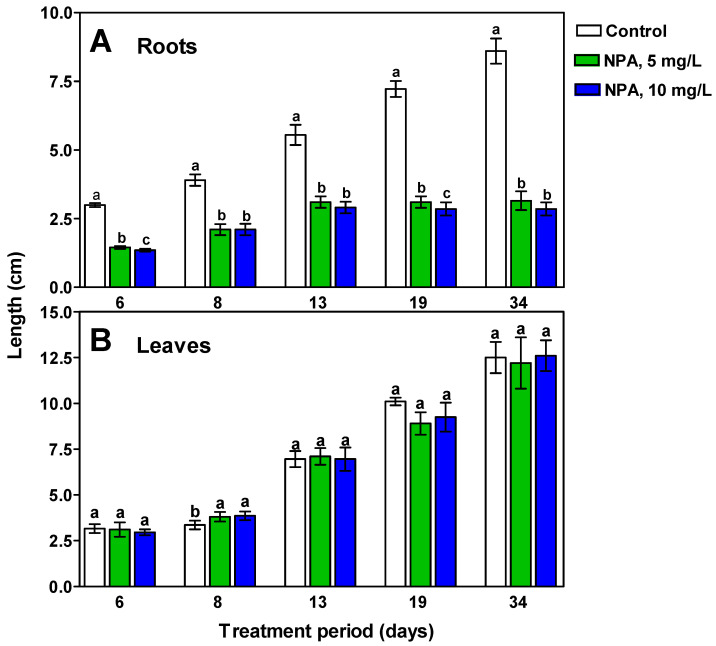
Results of root (**A**) and leaf (**B**) length measurements in *M. armeniacum* plants whose bulbs were kept in water or aqueous NPA solutions for the days indicated. Results (bars ± SD) on particular days of measurements marked with the same letter do not differ at a significance level of *p* = 0.05 according to Duncan’s test.

Continuous soaking of uncooled *M. armeniacum* bulbs in NPA solutions inhibited the elongation of roots, as well as changed their shapes and blocked their gravitropic response (Figure 1, Figure 2, Figure 3, Figure 4 and Figure 5). In addition, the applied NPA resulted in a significant promotion of root hairs. The inhibition of root growth by NPA was dose-dependent, but had no effect on leaf growth compared to *M. armeniacum* bulbs kept in water.

The root structure of *M. armeniacum* includes a stele of vascular tissues surrounded by a cortex composed of several layers of parenchyma cells and an outermost layer of rhizodermis/epiblema. The vascular system has no vascular cambium, resulting in a lack of secondary growth. The end of the root apical meristem is protected by the root cap. Immediately behind the root apex is the elongation zone, where cells undergo rapid division and elongation.

NPA significantly affected the morphology and anatomy of *M. armeniacum* roots (Figure 1). NPA clearly induced root swelling and increased both the number and length of root hairs near the root apex as well as in the elongation zone. Microscopic analyses of cross-sections and longitudinal sections of *M. armeniacum* roots showed significant differences in root architecture between roots from bulbs kept in water (control) and those treated with NPA solutions (Figure 2). This resulted in a marked thickening of the elongated part of the roots, which started to bend upwards (Figure 1C,F and Figure 3B).

In NPA-treated roots, apical meristem size, cortex cell thickness, rhizodermis thickness, root hairs length, cortex width, and root thickness increased. NPA, at the lower concentration, had little effect on the stele width, while a 10 mg/L NPA solution resulted in a significant increase in the stele width. In contrast, the use of NPA decreased cap thickness (Figure 2 and Figure 3). Cap thickness in control roots reached 180 μm, while in roots grown in a 10 mg/L NPA solution, it was around 100 μm (Figure 3B (bars 3)). In the terminal 2 cm sections of *M. armeniacum* roots growing in water, no root hairs were found, while in root samples growing in the presence of NPA, their lengths reached 130–170 μm (Figure 3B (bars 5)). Additionally, the thickness of roots growing in the presence of NPA at a concentration of 10 mg/L was twice that of those growing in water (Figure 3C (bars 8)).

Figure 4 shows the final effect caused by a 17-day treatment period in water (A), 5 mg/L (B), and 10 mg/L NPA solution (C) on the roots of *M. armenicaum.* An unnatural change in the growth direction of the roots can be observed (Figure 4B,C), as they turn upwards against the force of gravity.

Measurements of root and leaf length of *M. armeniacum* plants whose bulbs were treated with NPA for a given period indicate that the herbicide did not affect leaf growth, but inhibited root growth (Figure 5). During the initial 13 days of the experiment, there was a slight increase in root growth in NPA-treated bulbs, but their length was about half as long as that of control bulbs growing in water. After this period, the NPA-treated roots stopped growing completely, while there was a steady growth in the roots growing in water (Figure 5).

### 2.2. Content of Polar Compounds in M. armeniacum Roots

Both NPA aqueous solutions used had no effect on the organic acid and phosphate contents of *M. armeniacum* root tissues (Table 1). Similarly, the NPA solution had no effect on the levels of valine, alanine, proline, serine, asparagine, *iso*-leucine, aspartate, glutamate, glutamine, and *γ*-aminobutyric acid (GABA). In general, it can be concluded that the content of particular amino acids, as well as their totals in *M. armeniacum* roots, was lower after 17 days of the experiment than after 7 days.

The shorter period (7 days) of NPA treatment did not affect the content of known and unknown carbohydrates in the roots of *M. armenicaum* (Table 2; Figure S1). After a longer period (17 days), NPA decreased the content of fructose and *myo*-inositol and increased the content of sucrose and almost all unknown carbohydrates (Unk 2—Unk 7, probably fructans, i.e., fructosyl derivatives of glucose, GF2–GF4), as well as their total content.

### 2.3. Content of Phenolic Compounds in M. armeniacum Roots 

Caftaric acid and caffeic acid were, quantitatively, the main phenolic acids in the root tissues of *M. armeniacum* (Table 3). The content of free caffeic acid increased in root tissues growing in the presence of NPA solutions, while the content of its esters and glycosides decreased. The content of free caftaric acid and its esters also had a decreasing tendency, but not always statistically demonstrated. Caftaric acid is the product of the tartaric acid esterification by caffeic acid, and chicoric acid is the result of the tartaric acid esterification by two molecules of caffeic acid. The content of free ferulic acid and free *p*-coumaric acid, as well as their esters and glycosides, also decreased under the influence of NPA. The contents of other phenolic acids were very low, and their changes were not significant.

Flavonoid contents in the roots of *M. armeniacum* were very low, except catechin and apigenin (Table 4). NPA caused a marked reduction in the content of free apigenin as well as its esters and glycosides. In the case of catechin, there was also a decrease in its content under NPA, but this was not statistically confirmed due to the high value of the standard deviation.

In *M. armeniacum* root tissues, there were low contents of free cyanidin and pelargonidin, as well as the mono- and di-glucosides of cyanidin, pelargonidin, peonidin, and delphinidin (Table 5). Under the influence of NPA, there was a reduction in peonidin 3-glucoside, delphinidin di-glucoside, and pelargonidin-3-glucoside.

## 3. Discussion

One of the most well-known compounds that causes a reduction in auxin access or activity is NPA [9,10,11,12,13,14,15]. NPA caused growth inhibition of primary roots in *Arabidopsis thaliana*, mainly by reducing the rate of cell division [18]. Later, Chang et al. [23] found that grape leaf explants completely inhibited rooting in the presence of NPA at a concentration of 3 mg/L. Recently, we have demonstrated that application of NPA to the stem of *Solidago canadensis* induced a stress response in the form of stem swelling, accompanied by an increase in auxin and cytokinin levels [13]. This stress caused abnormal radial growth of the stem and changes in histological structure. Accumulation of auxins and cytokinins at a stem swelling area probably caused inhibition of their polar transport. Previously, we found that NPA induced local stem swelling in *Bryophyllum calycinum* plants and promoted rooting of shoot cuttings [14]. Furthermore, NPA, at the application area of the stem of *Bryophyllum calycinum,* caused a significant increase in IAA, cytokinins, and jasmonic acid levels. Also in the present study, NPA applied to the roots clearly caused them to swell, and increased both the number and length of root hairs (Figure 1, Figure 2 and Figure 3). Thus, it appears that the phenomenon of swelling in organs exposed to NPA is quite common.

It has been well demonstrated that auxin is important for many aspects of root development, including lateral root initiation and formation, root apical meristem formation, gravitropism, and root elongation [9,16,17,25,26,27,28]. The root cap is responsible for receiving the gravitational pulse, and its removal causes the root to become incapable of responding to the gravitational stimulus [25]. The auxin gradient in the root cap plays a key role in its development, while NPA disrupts this and induces growth incompatible with gravity [26]. The root cap functions as a local auxin sink, and its growth depends on the resulting auxin gradient [28,36]. Also, the application of NPA to *Arabidopsis thaliana* roots blocked their gravitational response, root waving and elongation, suppressed lateral root growth, and inhibited gravitropism [25,35,36,37,38]. Gravity sensing takes place in the root cap cells, where sedimentation of starch-filled amyloplasts triggers a pathway that results in the relocation to the lower half of the cell of PIN proteins that facilitate auxin efflux. This causes auxin accumulation in the lower half of the root and bending of the root tip in the elongation zone [39,40].

Roots are a good model for investigating the mechanisms of gravitropism, as it has been shown that there are separate gravity-sensing sites (the root cap) and a curvature response area (the elongation zone), and deleting the root cap results in a loss of root gravitropism [39]. For example, tomato root growth was inhibited by NPA and caused a 90-degree change in root orientation from vertical [40].

The results of our current study confirm previous reports, which indicated that transport limitations and/or auxin distribution by NPA cause a loss of gravity sensing by root tips [25,26]. In the mentioned papers, NPA applied to the roots of *Arabidopsis thaliana* blocked the gravity response, root waving, and root elongation. The thickening of the root meristem observed during our experiments, visible both on microscopic images and on cross-sections of roots, may indicate a disturbance in auxin transport or allocation (Figure 1, Figure 2 and Figure 3). MS/MS scanning performed on *M. armeniacum* root extracts showed the presence of IAA in control samples, but its level was below the detection limit in roots grown in the presence of both NPA solutions. This indicates an inhibition of the polar transport of this phytohormone or its reversible or irreversible inactivation. However, this supposition requires further detailed study.

As a result of the NPA’s impact, the growth of the roots in line with the direction of gravity was disrupted, and they began to bend upwards (Figure 4). This was accompanied by significant increases in apical meristem size, an increase in cortex cells, rhizodermis thickness, length of root hairs, width of stele, width of cortex, and root thickness. However, the application of NPA reduced the cap thickness and had little or no effect on the size of the stele (Figure 3). Probably all the effects of NPA were related to the disruption of auxin transport, as was previously demonstrated [23,24,25,26,27,28].

Carbohydrates are the primary source of carbon and energy, and regulate gene expression [41,42]. Auxin, produced in young shoot tissues, promotes root development by controlling carbohydrate import into sink organs [43]. This process critically affects plant growth and development [44,45,46]. The interaction between carbohydrates and auxins plays an important role in both cell division and growth [47,48]. Auxin stimulates carbohydrate mobilization in leaf tissues and increases carbohydrate translocation towards sink organs, including roots [48]. It was found that starch-filled amyloplasts present in root tips settle down under the influence of gravity because the starch has a density of 1.5 g/cm^3^, while the surrounding cytoplasm has a density below 1.1 g/cm^3^ [36,38]. The results of the root carbohydrate analyses obtained in the current study indicate increased accumulation of low-molecular-weight carbohydrates, presumably fructans, in roots growing in the presence of NPA (Table 2). This may indicate an induction of polyfructan hydrolysis. Whether the changes in polyfructan levels in *M. armeniacum* roots in response to NPA are related to root gravitropism is an open question and worth undertaking further research in this direction.

The root tips are covered with a cap, which protects the root from soil obstacles, and above the cap is the root elongation, where cell division, initial cell differentiation, and growth take place. The reduction in root size and meristem thickening shown in our study may be another symptom or cause of a disturbance in the direction of root growth (Figure 1, Figure 2 and Figure 3).

The results of several studies indicate the involvement of flavonols in controlling root gravitropism by affecting auxin transport, which is essential for gravitropism [49,50,51,52,53]. Flavonols have been shown to regulate plant growth, development, and physiology through two distinct mechanisms: maintenance of reactive oxygen species (ROS) homeostasis and inhibition of auxin transport [52]. *Natural flavonols can compete with the synthetic* inhibitor of auxin transport, NPA, by binding to proteins involved in auxin transport [54,55]. In the roots of M. armeniacum, the content of flavonols (quercetin, kaempferol, and luteolin) was very low (Table 4), which may indicate that their role in reducing auxin transport is less significant or unimportant. Also, catechin content was decreased under the influence of NPA, which also does not indicate its important role in this process. However, it has previously been shown that flavonols, especially quercetin, act as negative regulators of root hair formation in Arabidopsis [54]. Flavonols in root hairs act by controlling the levels of reactive oxygen species. Similarly, in a tomato mutant that has reduced flavonol levels, there is an increased number of root hairs [56]. The increase in number and length of root hairs observed in our study under the influence of NPA (Figure 1 and Figure 3) may therefore be related to a reduction in the levels of flavonols and catechin or other polyphenols (Table 4). On the other hand, there are speculations that the inhibition of the accumulation of polyphenolic compounds in the roots is caused by high IAA oxidase activity, which increases the growth rate of the upper part of the roots and interferes with their gravitropic response [57,58]. The reduction in many phenolic compounds observed in our study under the influence of NPA (Table 3 and Table 4) indicates that this may be one of the symptoms/causes of root growth disturbances. However, this supposition requires further research.

## 4. Materials and Methods

### 4.1. Plant Material

Uncooled bulbs of grape hyacinth (*Muscari armeniacum* Leichtl. Ex. Baker) with a circumference of 3–4 cm were used for the study. In the initial experiment, the bulbs were kept in a Petri dish filled with distilled water (control) or NPA (TCI, Osaka, Japan) solutions from 5 to 80 mg/L. During the experiment, morphological observations, as well as root and leaf measurements and pictures, were taken. In further experiments, the roots developed from *M. armeniacum* bulbs soaked in water (control) or NPA (5 or 10 mg/L) were used for histological examinations, while other root tissues, after freeze-drying and grinding, were used for the analysis of primary and secondary metabolite content. For the determination of metabolites in the roots, their samples were taken after 7 and/or 17 days of the experiment duration. All experiments were conducted at temperatures of 18–22 °C under natural greenhouse light and carried out in August and September 2024.

### 4.2. Histological Observations and Measurements

Roots from bulbs, after a 9-day growth period in water (control) or in NPA at concentrations of 5 mg/L and 10 mg/L, were used for histological examination. Root tip fragments of 2 cm length were fixed in a solution containing chromic acid, acetic acid, and formalin (CrAF) for 48 h and subsequently embedded in paraffin, following the method described earlier [59]. Their longitudinal and cross-sections, each 10 μm in thickness, were prepared using a rotary microtome (Leica, Wetzlar, Germany) and stained with 1% safranin and 0.5% fast green. The obtained sections were mounted in Canada balsam (Sigma-Aldrich, Merck, Burlington, MA, USA) and examined under a light microscope equipped with polarization capabilities (Eclipse 80i with NIS-Elements BR ver. 4.00 imaging software, Nikon Instruments Inc., Tokyo, Japan). The root diameter, the thickness of the root cortex in the elongation zone, and the dimensions of the cells inside the root cortex were measured in the samples thus obtained.

### 4.3. Determination of Organic Acids, Amino Acids, and Soluble Carbohydrates

Primary polar metabolites were extracted from powdered freeze-dried samples with a mixture of methanol/water (1:1, *v*/*v*), containing ribitol as an internal standard. The homogenates were heated at 70 °C with continuous shaking (Thermo-shaker MS-100 ALLSHENG, Hangzhou, China) for 30 min. After centrifugation, the supernatant was extracted with chloroform to remove lipidic substances, and the upper layer (methanol/water polar fraction) was dried in a speed vacuum rotary evaporator (JWElectronic, Warsaw, Poland). The dry residues were derivatized in two steps using O-methoxyamine hydrochloride in pyridine (Sigma-Aldrich, Merck, Burlington, MA, USA) and a mixture of *N*-methyl-*N*-(trimethylsilyl)-trifluoroacetamide in pyridine (Sigma-Aldrich, Merck, Burlington, MA, USA). All further details of the method of analysis of polar metabolites have been described previously [60,61].

Qualitative and quantitative analyses of the metabolites were performed using a gas chromatograph GC-2010 (Shimadzu, Kyoto, Japan). For the separation of a mixture of metabolite derivatives, the capillary column ZEBRON ZB5-MSi (length, 30 m, diameter 0.25 mm, film thickness 0.25 μm, 5% phenyl–95% dimethylpolysiloxane, Phenomenex, Torrance, CA, USA) was used. Further details of the GC conditions have been described previously [62]. The identification of metabolites was confirmed by gas chromatography coupled with mass spectrometry (GCMS-QP2010 Shimadzu, Kyoto, Japan). Data were collected and analyzed using GCMS Solution 2.50 SU3 software (Shimadzu, Japan). Polar metabolites were identified by comparing the retention indices and mass spectra collected in the NIST 05 library (Shimadzu, Kyoto, Japan) and the internal collection of mass spectra obtained for the original standards purchased from SIGMA [62].

Soluble carbohydrates were extracted from powdered, freeze-dried samples with a mixture of ethanol/water (1:1, *v*/*v*), containing xylitol as an internal standard. The homogenates were heated at 90 °C with continuous shaking, then centrifuged (24,000× *g* at 4 °C for 20 min), and a part of the supernatant was purified on centrifuge filters (PVDF, 0.2 μm, Thermo Fischer Scientific, Rockwood, TN, USA), and then dried in a of carbohydrates speed vacuum rotary evaporator. Dry residues were derivatized in a mixture of 1-(trimethylsilyl)imidazole (TMSI) with pyridine (1:1), at 80 °C for 45 min. The TMS-derivatives were separated on the capillary column ZEBRON ZB-1 (length 15 m, diameter 0.25 mm, film thickness 0.10 μm, 100% dimethylpolysiloxane, Phenomenex, Torrance, CA, USA) [63].

The concentration of metabolites analyzed by the GC-FID method was calculated from standard curves obtained for the appropriate original metabolites using the internal standard method, except for the unknown carbohydrates. Unknown 1 was calculated on the basis of the *myo*-inositol standard, unknown 2 based on the raffinose standard (galactosyl-sucrose), unknowns 3 and 4 on the basis of the stachyose standard (di-galactosyl-sucrose), and unknowns 5, 6, and 7 on the basis of the verbascose standard (tri-galactosyl-sucrose). Their preliminary identification was carried out by comparing the retention times of root tissue components to those of sucrose galactosides with a similar degree of polymerization (Figure S1).

### 4.4. Determination of Phenolic Compounds

The content of phenolic acids and flavonoids was determined according to the method described in detail by Dębski et al. [64]. Briefly, a crude extract was obtained from freeze-dried plant samples by stirring overnight with a mixture of methanol, water, and formic acid (80:19.9:0.1, *v*/*v*/*v*). The extraction was repeated five times, and the obtained crude extracts were collected. The free forms of phenolic acids and flavonoids were then isolated with diethyl ether. Next, after the free forms were isolated, esters present in the extracts were hydrolyzed with 4 M NaOH. Subsequently, glycosides present in the extracts were hydrolyzed in the residues with 6 M HCl. After each step, the released compounds were isolated with diethyl ether, and the ether was evaporated to dryness under a stream of nitrogen. The free compounds and compounds released from bound forms were dissolved in methanol, centrifuged and subjected to analysis on an HPLC–MS/MS system (QTRAP 5500 ion trap mass spectrometer, AB SCIEX, Concord, ON, Canada) equipped with a HALO C18 column (2.7 μm particles, 0.5 × 50 mm, Eksigent, St. Markham, ON, Canada) at 45 °C, with a flow rate of 0.015 mL/min. The gradient of the elution solvents A (water/formic acid, 99.05/0.95; *v*/*v*) and B (acetonitrile/formic acid, 99.05/0.95, *v*/*v*) was as follows: 5% B for 0.1 min, 90–95% B in 1.9 min, 90% B for 0.5 min, 90–5% B in 0.2 min, and 5% B for 0.3 min. The contents of phenolic compounds obtained by acid and alkaline hydrolysis were presented as their free, ester, and glycosidic forms or as a total, i.e., the sum of all forms.

### 4.5. Determination of Anthocyanins

The extraction of anthocyanins and the determination of their content were carried out using a method described in detail by Wiczkowski et al. [65]. Briefly, freeze-dried and powdered samples were extracted with 0.4% trifluoroacetic acid in methanol by vortexing and sonication, and the obtained extracts were centrifuged, and the supernatants were combined. The analyses were carried out using an LC-200 Eksigent HPLC system coupled with a Triple TOF 5600 + mass spectrometer (AB SCIEX, Vaughan, ON, Canada). Chromatographic separation of anthocyanins was carried out on the HALO C18 column (2.7 μm, 100 × 0.5 mm, Eksigent, Vaughan, ON, Canada) with a solvent gradient system consisting of solvent A (0.95% formic acid aqueous solution) and solvent B (0.95% formic acid in acetonitrile). Identification of the anthocyanins was based on a comparison of their retention time and MS/MS fragmentation spectrum (m/z values) with data from standards analysis, the published data, and on the interpretation of the fragmentation spectrum obtained. Further details of the method of anthocyanin analysis are described in the cited paper [65].

### 4.6. Statistical Evaluation of Results

Results of measurements were subjected to analysis of variance (ANOVA), followed by Duncan’s multiple range test. For statistical analysis, three replicates were used (each replicate consisted of 20 measurements for histological measurements), and 20 to 100 mg of freeze-dried plant samples for analyses of primary and secondary metabolites. *P* values of < 0.05 were considered to be statistically significant for means.

## 5. Conclusions

In the present study, we have shown that treatment of uncooled *Muscari armeniacum* bulbs with an aqueous solution of 1-*N*-naphthylphthalamic acid (NPA) affected root growth and development, as well as the levels of many metabolites. In roots developing under these conditions, growth has been inhibited, and the elongation part has been swollen. There have also been changes in the size of different parts of the root and a disruption of their gravitropic direction of growth, as the roots began to bend upwards against the direction of gravity. Such symptoms have not previously been found in *M. armeniacum.* However, the application of NPA did not affect leaf growth as well as the content of most amino acids, organic acids, and some carbohydrates in the roots, but increased the levels of unknown saccharides, probably oligofructans. The effect of NPA on the decrease in levels of many phenolic compounds observed in the present study may indicate that this may be one of the symptoms/causes of root growth disorders and may have affected the increase in the number and length of root hairs.

## Figures and Tables

**Table 1:** Organic acid, phosphate, and amino acid contents (mg/g dry weight) in *M. armeniacum* roots after their 7-day and 17-day treatment period in water or aqueous NPA solutions. * The means in the rows marked with the same letter do not differ at the significance level of *p* = 0.05 according to Duncan’s test.

Analyzed Compound	7-Day Treatment Period			17-Day Treatment Period		
	Control, Water	NPA, 5 mg/L	NPA, 10 mg/L	Control, Water	NPA, 5 mg/L	NPA, 10 mg/L
Succinate	0.43 ± 0.05 ^a^*	0.43 ± 0.05 ^a^	0.42 ± 0.01 ^a^	0.24 ± 0.01 ^b^	0.26 ± 0.03 ^b^	0.24 ± 0.03 ^b^
Fumarate	0.12 ± 0.03 ^a^	0.13 ± 0.05 ^a^	0.16 ± 0.03 ^a^	0.06 ± 0.01 ^a^	0.10 ± 0.01 ^a^	0.08 ± 0.02 ^a^
Malate	33.07 ± 4.46 ^a^	34.65 ± 3.58 ^a^	33.81 ± 0.25 ^a^	21.05 ± 0.95 ^b^	22.64 ± 0.72 ^b^	22.09 ± 0.50 ^b^
Citrate	10.81 ± 2.55 ^a^	7.99 ± 2.15 ^a^	9.77 ± 0.34 ^a^	7.49 ± 0.67 ^a^	7.34 ± 0.80 ^a^	8.68 ± 4.05 ^a^
Total acids	44.4 3± 6.95 ^ab^	43.20 ± 4.47 ^ab^	44.16 ± 0.56 ^a^	28.84 ± 0.80 ^c^	30.33 ± 1.45 ^bc^	31.09 ± 3.62 ^bc^
Phosphate	4.69 ± 0.69 ^a^	4.94 ± 0.53 ^a^	4.70 ± 0.26 ^a^	3.96 ± 0.17 ^a^	4.56 ± 0.16 ^a^	4.23 ± 0.15 ^a^
Valine	0.19 ± 0.07 ^a^	0.21 ± 0.09 ^a^	0.16 ± 0.10 ^a^	0.13 ± 0.01 ^a^	0.07 ± 0.02 ^a^	0.07 ± 0.02 ^a^
Alanine	1.33 ± 0.19 ^a^	1.32 ± 0.13 ^a^	1.20 ± 0.09 ^a^	0.73 ± 0.05 ^b^	0.90 ± 0.01 ^b^	0.85 ± 0.31 ^ab^
Proline	1.09 ± 0.05 ^a^	1.29 ± 0.27 ^ab^	1.22 ± 0.13 ^ab^	0.72 ± 0.09 ^b^	0.88 ± 0.01 ^b^	0.64 ± 0.23 ^ab^
Serine	0.80 ± 0.14 ^a^	0.73 ± 0.14 ^a^	0.70 ± 0.09 ^a^	0.44 ± 0.04 ^a^	0.46 ± 0.05 ^a^	0.39 ± 0.09 ^a^
Aspartate	0.51 ± 0.07 ^a^	0.44 ± 0.05 ^a^	0.37 ± 0.05 ^ab^	0.29 ± 0.03 ^ab^	0.26 ± 0.01 ^b^	0.28 ± 0.01 ^b^
Glutamate	0.49 ± 0.13 ^ab^	0.88 ± 0.11 ^a^	0.67 ± 0.01 ^a^	0.31 ± 0.02 ^b^	0.57 ± 0.04 ^ab^	0.44 ± 0.13 ^ab^
Asparagine	6.67 ± 1.14 ^ab^	7.83 ± 1.49 ^ab^	7.41 ± 0.44 ^a^	4.54 ± 0.29 ^b^	5.34 ± 0.73 ^ab^	4.56 ± 1.31 ^ab^
*iso*-Leucine	0.28 ± 0.05 ^ab^	0.32 ± 0.05 ^ab^	0.27 ± 0.01 ^a^	0.17 ± 0.01 ^b^	0.22 ± 0.03 ^ab^	0.19 ± 0.05 ^ab^
OH-Proline	0.55 ± 0.19 ^abc^	0.68 ± 0.09 ^a^	0.48 ± 0.08 ^ab^	0.34 ± 0.04 ^b^	0.21 ± 0.02 ^b^	0.10 ± 0.01 ^c^
GABA	0.24 ± 0.04 ^a^	0.29 ± 0.01 ^a^	0.24 ± 0.02 ^a^	0.25 ± 0.03 ^a^	0.33 ± 0.01 ^a^	0.33 ± 0.09 ^a^
Glutamine	0.05± 0.02 ^a^	0.10 ± 0.03 ^a^	0.11 ± 0.02 ^a^	0.04 ± 0.01 ^a^	0.10 ± 0.02 ^a^	0.09 ± 0.04 ^a^
Total amino acids	12.19 ± 1.92 ^ab^	14.10 ± 2.13 ^ab^	12.84 ± 0.82 ^a^	7.98 ± 0.55 ^b^	9.34 ± 0.89 ^ab^	7.93 ± 1.79 ^ab^

**Table 2:** Carbohydrate contents (mg/g dry weight) in *M. armeniacum* roots after their 7-day and 17-day treatment period in water or aqueous NPA solutions. * The means in the rows marked with the same letter do not differ at the significance level of *p* = 0.05 according to Duncan’s test. Unk 1–Unk 7 means unknown carbohydrates.

Analyzed Carbohydrate	7-Day Treatment Period			17-Day Treatment Period		
	Control, Water	NPA, 5 mg/L	NPA, 10 mg/L	Control, Water	NPA, 5 mg/L	NPA, 10 mg/L
Fructose	50.12 ± 2.86 ^c^*	36.67 ± 1.34 ^d^	39.28 ± 2.42 ^cd^	80.75 ± 2.14 ^a^	84.24 ± 2.42 ^a^	70.83 ± 1.51 ^b^
Glucose	68.61 ± 2.29 ^b^	62.04 ± 9.13 ^b^	60.08 ± 4.32 ^b^	91.49 ± 2.72 ^a^	98.32 ± 2.56 ^a^	107.0 ± 8.34 ^a^
*myo*-Inositol	9.79 ± 0.61 ^a^	9.27 ± 0.60 ^a^	9.65 ± 0.72 ^a^	4.58 ± 0.20 ^b^	5.15 ± 0.18 ^b^	5.38 ± 0.22 ^b^
Sucrose	49.89± 3.94 ^c^	57.59 ± 4.02 ^c^	54.93 ± 3.98 ^c^	60.36 ± 0.84 ^c^	73.31 ± 2.03 ^b^	82.91 ± 1.51 ^a^
Unk 1	3.41 ± 0.19 ^ab^	2.80 ± 0.11 ^b^	2.85 ± 0.17 ^b^	3.49 ± 0.03 ^ab^	4.00 ± 0.24 ^a^	4.21 ± 0.62 ^a^
Unk 2 (GF2)	45.54 ± 3.28 ^abc^	49.97 ± 3.72 ^a^	50.27 ± 4.52 ^abc^	38.71 ± 0.46 ^c^	44.72 ± 1.57 ^b^	55.58 ± 1.94 ^a^
Unk 3 (GF3)	16.53 ± 1.47 ^bc^	20.68 ± 2.00 ^ab^	21.18 ± 2.25 ^ab^	14.34 ± 0.14 ^c^	17.62 ± 0.64 ^b^	22.11 ± 0.63 ^a^
Unk 4 (GF3)	20.30 ± 1.68 ^a^	20.58 ± 2.09 ^a^	21.19 ± 2.17 ^a^	11.81 ± 0.20 ^c^	11.72 ± 0.49 ^c^	14.54 ± 0.41 ^b^
Unk 5 (GF4)	5.84 ± 0.69 ^ab^	8.49 ± 1.07 ^a^	8.84 ± 1.17 ^a^	4.76 ± 0.06 ^b^	5.85 ± 0.23 ^a^	7.10 ± 0.10 ^a^
Unk 6 (GF4)	0.79 ± 0.26 ^b^	1.55 ± 0.24 ^ab^	1.50 ± 0.26 ^ab^	1.24 ± 0.03 ^b^	2.26 ± 0.15 ^a^	2.56 ± 0.05 ^a^
Unk 7 (GF4)	14.88 ± 1.09 ^ab^	17.28 ± 1.98 ^a^	18.75 ± 2.42 ^a^	9.13 ± 0.13 ^c^	9.76 ± 0.33 ^c^	11.98 ± 0.19 ^b^
Total unk 1–7	107.3 ± 8.01 ^a^	121.4 ± 10.9 ^a^	124.6 ± 12.6 ^a^	83.47 ± 0.73 ^b^	95.9 ± 3.6 ^ab^	118.1 ± 3.0 ^a^
Total carbohydrates	285.7 ± 16.4 ^b^	286.9 ± 24.6 ^b^	288.5 ± 23.5 ^b^	320.6 ± 4.1 ^b^	356.9 ± 10.7 ^ab^	384.2 ± 12.0 ^a^

**Table 3:** Phenolic acid contents (μg/g dry weight ± SD) in *M. armeniacum* roots after their 17-day treatment period in water or aqueous NPA solutions. * The means in the rows marked with the same letter do not differ at the significance level of *p* = 0.05 according to Duncan’s test. tr**—means traces, *i.e*., below 0.01 μg/g dry weight.

Acid Forms	Control, Water	NPA, 5 mg/L	NPA, 10 mg/L
Ferulic acid (3-methoxy-4-hydroxycinnamic acid)			
Free	10.75 ± 0.02 ^a^*	6.68 ± 0.46 ^b^	5.26 ± 0.54 ^b^
Esters	5.79 ± 0.05 ^a^	3.28 ± 0.15 ^c^	4.07 ± 0.09 ^b^
Glycosides	3.50 ± 0.52 ^a^	0.43 ± 0.02 ^b^	0.34 ± 0.11 ^b^
Total	20.04 ± 0.59 ^a^	10.39 ± 0.63 ^b^	9.67 ± 0.74 ^b^
Sinapic acid (3,5-dimethoxy-4-hydroxycinnamic acid)			
Free	tr**	tr	tr
Esters	0.24 ± 0.04 ^a^	0.17 ± 0.03 ^a^	0.09 ± 0.02 ^a^
Glycosides	tr	tr	tr
Total	0.24 ± 0.04 ^a^	0.17 ± 0.03 ^a^	0.09 ± 0.02 ^a^
*p*-Coumaric acid (*trans*-4-hydroxycinnamic acid)			
Free	0.36 ± 0.01 ^a^	0.13 ± 0.02 ^b^	0.11 ± 0.02 ^b^
Esters	1.75 ± 0.04 ^a^	0.92 ± 0.04 ^b^	0.92 ± 0.01 ^b^
Glycosides	0.38 ± 0.01 ^a^	0.03 ± 0.01 ^b^	0.03 ± 0.01 ^b^
Total	2.49 ± 0.05 ^a^	1.08 ± 0.07 ^b^	1.07 ± 0.03 ^b^
Protocatechuic acid (3,4-dihydroxybenzoic acid)			
Free	0.09 ± 0.05 ^a^	0.04 ± 0.03 ^a^	0.08 ± 0.01 ^a^
Esters	0.04 ± 0.01^a^	0.03 ± 0.00 ^a^	0.03 ± 0.00 ^a^
Glycosides	0.03 ± 0.00 ^a^	0.04 ± 0.01 ^a^	0.03 ± 0.00 ^a^
Total	0.16 ± 0.05 ^a^	0.11 ± 0.03 ^a^	0.14 ± 0.01^a^
Ellagic acid			
Free	1.16 ± 0.17 ^a^	1.00 ± 0.35 ^a^	0.76 ± 0.19 ^a^
Esters	0.26 ± 0.02 ^a^	0.17 ± 0.01 ^a^	0.15 ± 0.01 ^a^
Glycosides	0.59 ± 0.07 ^a^	0.61 ± 0.12 ^a^	0.48 ± 0.06 ^a^
Total	2.01 ± 0.27 ^a^	1.77 ± 0.47 ^a^	1.39 ± 0.25 ^a^
Caffeic acid			
Free	38.85 ± 1.39 ^c^	54.41 ± 0.22 ^a^	49.50 ± 1.06 ^b^
Esters	3.56 ± 0.03 ^a^	2.74 ± 0.13 ^b^	2.04 ± 0.23 ^b^
Glycosides	6.72 ± 1.00 ^a^	1.03 ± 0.22 ^b^	1.29 ± 0.13 ^b^
Total	49.12 ± 2.42 ^b^	58.19 ± 0.87 ^a^	52.83 ± 1.41^b^
Caftaric acid (2-caffeoyl-L-tartaric acid)			
Free	42.17 ± 1.14 ^a^	30.32 ± 1.61 ^b^	37.48 ± 4.83 ^ab^
Esters	3.53 ± 0.91 ^a^	2.58 ± 0.25 ^a^	2.73 ± 0.11 ^a^
Glycosides	tr	tr	tr
Total	45.69 ± 2.06 ^a^	32.90 ± 1.86 ^b^	40.21 ± 4.94 ^ab^
Chicoric acid (2,3-dicaffeoyl-L-tartaric acid)			
Free	0.09 ± 0.02 ^a^	0.03 ± 0.01 ^a^	0.17 ± 0.02 ^a^
Esters	tr	tr	tr
Glycosides	0.01 ± 0.00 ^b^	0.12 ± 0.01 ^a^	0.04 ± 0.02 ^b^
Total	0.10 ± 0.02 ^a^	0.15 ± 0.01 ^a^	0.20 ± 0.04 ^a^

**Table 4:** Flavonoid contents (μg/g dry weight ± SD) in *M. armeniacum* roots after their 17-day treatment period in water or aqueous NPA solutions. * The means in the rows marked with the same letter do not differ at the significance level of *p* = 0.05 according to Duncan’s test. tr**—means traces, *i.e*., below 0.01 μg/g dry weight.

Flavonoid Forms	Control, Water	NPA, 5 mg/L	NPA, 10 mg/L
Quercetin			
Free	tr	0.10±0.01	tr
Esters	tr**	tr	tr
Glycosides	0.05 ± 0.01 ^a^*	0.03 ± 0.02 ^a^	0.02 ± 0.02 ^a^
Total	0.05 ± 0.01^a^	0.13 ± 0.03 ^a^	0.02 ± 0.02 ^a^
Apigenin			
Free	1.60 ± 0.08 ^a^	0.67 ± 0.06 ^b^	0.41 ± 0.01 ^c^
Esters	0.07 ± 0.01	tr	tr
Glycosides	2.01 ± 0.04 ^a^	0.14 ± 0.02 ^b^	0.08 ± 0.01 ^b^
Total	3.67 ± 0.13 ^a^	0.81 ± 0.06 ^b^	0.49 ± 0.01 ^c^
Kaempferol			
Free	tr	tr	tr
Esters	tr	tr	tr
Glycosides	tr	tr	tr
Total	tr	tr	tr
Luteolin			
Free	0.03 ± 0.01	tr	tr
Esters	tr	tr	tr
Glycosides	0.16 ± 0.02 ^a^	0.06 ± 0.01 ^b^	0.05 ± 0.01 ^b^
Total	0.19 ± 0.03 ^a^	0.06 ± 0.01 ^b^	0.05 ± 0.01 ^b^
Naringenin			
Free	0.06 ± 0.02 ^a^	0.03 ± 0.01 ^a^	0.02 ± 0.01 ^a^
Esters	tr	tr	tr
Glycosides	0.19 ± 0.05	tr	tr
Total	0.25 ± 0.06 ^a^	0.03 ± 0.01 ^b^	0.02 ± 0.01 ^b^
Catechin			
Free	42.85 ± 5.56 ^a^	28.05 ± 4.58 ^a^	32.54 ± 0.07 ^a^
Esters	21.85 ± 3.06 ^a^	12.23 ± 1.64 ^a^	13.69 ± 2.93 ^a^
Glycosides	2.58 ± 1.11 ^b^	5.88 ± 0.31 ^a^	6.92 ± 0.33 ^a^
Total	67.28 ± 9.73 ^a^	46.16 ± 6.53 ^a^	53.16 ± 3.32 ^a^

**Table 5:** Anthocyanin contents (μg/g dry weight ± SD) in *M. armeniacum* roots after their 17-day treatment period in water or aqueous NPA solutions. * The means in the rows marked with the same letter do not differ at the significance level of *p* = 0.05 according to Duncan’s test. tr**—means traces, *i.e*., below 0.01 μg/g dry weight.

Anthocyanin	Control, Water	NPA, 5 mg/L	NPA, 10 mg/L
Cyanidin	tr**	tr	tr
Cyanidin 3-glucoside	0.10 ± 0.02 ^a^*	0.11 ± 0.08 ^a^	0.15 ± 0.07 ^a^
Cyanidin diglucoside	0.16 ± 0.01 ^a^	0.13 ± 0.01 ^a^	0.14 ± 0.01 ^a^
Peonidin diglucoside	tr	tr	tr
Peonidin 3-glucoside	0.40 ± 0.03 ^a^	0.18 ± 0.01 ^b^	0.13 ± 0.01 ^b^
Delphinidin diglucoside	0.38 ± 0.02 ^a^	0.22 ± 0.01 ^b^	0.19 ± 0.02 ^b^
Delphinidin 3-glucoside	0.05 ± 0.01 ^a^	0.04 ± 0.01 ^a^	0.03 ± 0.01 ^a^
Pelargonidin	tr	tr	tr
Pelargonidin 3-glucoside	1.43 ± 0.03 ^a^	0.61 ± 0.04 ^b^	0.61 ± 0.01 ^b^

## Data Availability

The data obtained and used in this study are available in the manuscript.

## Supplementary Materials

The following supporting information can be downloaded at https://www.mdpi.com/article/10.3390/ijms26136431/s1.

## Abbreviations

The following abbreviations are used in this manuscript:

NPA	1-*N*-naphthylphthalamic acid
ABA	Abscisic acid
IAA	Indolyl-*3*-acetic acid
GA	Gibberellic acid
BA	Benzyladenine
PIN	Proteins that facilitate auxin efflux
GC-MS	Gas chromatography-mass spectrometry
HPLC-MS	High-performance liquid chromatography-mass spectrometry
SD	Standard deviation
