# Supplementary material for: Effects of 1-N-Naphthylphthalamic Acid on Root and Leaf Development of Muscari armeniacum and the Related Metabolic and Physiological Features
Source: Int J Mol Sci. 2025 Jul 3;26(13):6431. doi: 10.3390/ijms26136431 (PMC12249636; doi:10.3390/ijms26136431)
Supplement: Supplementary file 1 [file ijms-26-06431-s001.zip › ijms-3699715-supplementary.pdf]

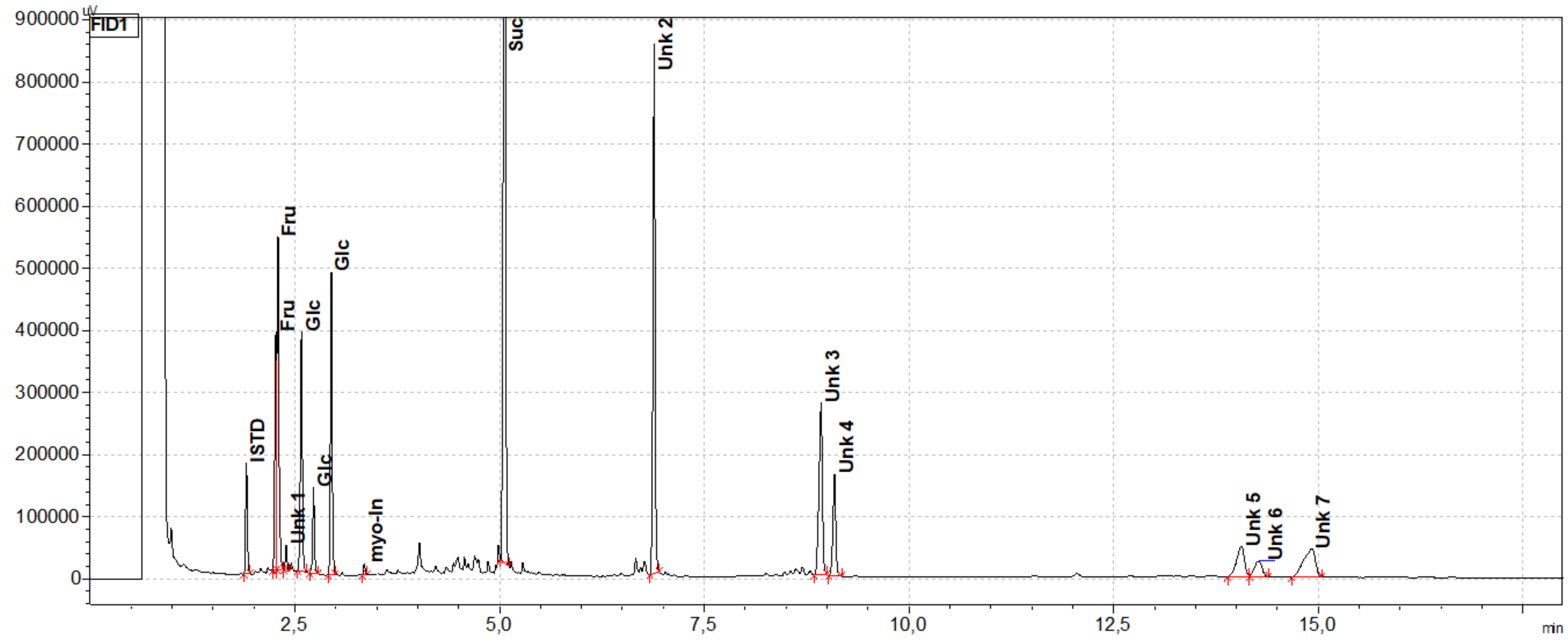

Figure S1. The gas chromatogram of TMS-derivatives of soluble carbohydrates extracted from roots of *Muscari armeniacum*. Abbreviations: ISTD – internal standard (xylitol), Fru – fructose, Glc – glucose, myo-In – *myo*-Inositol, Suc – sucrose, Unk 1 – unknown monosaccharide, Unk 2-7 – unknown sugars, presumably *tri*- (Unk 2), *tetra*- (Unk 3 and 4) and *penta*-saccharides (Unk 5-7).
